# Circadian rhythm and circulating cell-free DNA release on healthy subjects

**DOI:** 10.1038/s41598-023-47851-w

**Published:** 2023-12-07

**Authors:** Geoffroy Poulet, Jean-Sébastien Hulot, Anne Blanchard, Damien Bergerot, Wenjin Xiao, Frederic Ginot, Audrey Boutonnet-Rodat, Abdelli Justine, Guillaume Beinse, Vanna Geromel, Laurence Pellegrina, Michel Azizi, Pierre Laurent-Puig, Leonor Benhaim, Valerie Taly

**Affiliations:** 1 Université de Paris, UMR-S1138, CNRS SNC5096, Équipe Labélisée Ligue Nationale Contre le Cancer, Centre de Recherche des Cordeliers, Paris, France; 2 Eurofins-Biomnis, Gerland, Lyon, France; 3 CIC1418 and DMU CARTE, AP-HP, Hôpital Européen Georges-Pompidou, 75015 Paris, France; 4 Adelis, 478 Rue de la Découverte, 31670 Labège, France; 5 Department of Visceral and Surgical Oncology, Gustave Roussy, Villejuif, France; 6 Biochemistry Department - Unit of Pharmacogenetic and Molecular Oncology, Hôpital Européen Georges Pompidou (HEGP), Assistance Publique Hôpitaux de Paris (AP-HP), Paris, France

**Keywords:** Biological techniques, Cancer, Biomarkers, Oncology, Chemistry

## Abstract

In the last decade, clinical studies have investigated the clinical relevance of circulating cell-free-DNA (ccfDNA) as a diagnostic and prognosis tool in various diseases including cancers. However, limited knowledge on ccfDNA biology restrains its full development in the clinical practice. To improve our understanding, we evaluated the impact of the circadian rhythm on ccfDNA release in healthy subjects over a 24-h period. 10 healthy female subjects underwent blood sampling at 8am and 20 healthy male subjects underwent serial blood sampling (8:00 AM, 9:00 AM, 12:00 PM, 4:00 PM, 8:00 PM, 12:00 AM, 4 AM (+ 1 Day) and 8 AM (+ 1 Day)). We performed digital droplet-based PCR (ddPCR) assays to target 2 DNA fragments (69 & 243 bp) located in the *KRAS* gene to determine the ccfDNA concentration and fragmentation profile. As control, half of the samples were re-analyzed by capillary miniaturized electrophoresis (BIAbooster system). Overall, we did not detect any influence of the circadian rhythm on ccfDNA release. Instead, we observed a decrease in the ccfDNA concentration after meal ingestion, suggesting either a post-prandial effect or a technical detection bias due to a higher plasma load in lipids and triglycerides. We also noticed a potential effect of gender, weight and creatinine levels on ccfDNA concentration.

## Introduction

The presence of fragmented circulating cell-free DNA (ccfDNA) within peripheral blood was first described by Mandel & Metais in 1948^1^. The medical community has shown great interest in ccfDNA analyses across various disciplines, including infectiology, obstetrics, transplantation, surgery and oncology. In 1989, Stroun et al.^2^ opened the door to numerous applications when they demonstrated that, in cancer patients, a fraction of ccfDNA corresponded to cell-free tumor DNA (ctDNA). Since then, the utility of ccfDNA detection has been largely explored for cancer screening, diagnosis and prognosis^3–5^ through the analysis of somatic mutations^5,6^, chromosomal alterations^7^ and epigenetic changes^8^. More recently, several teams demonstrated the pertinence of ctDNA analysis to detect post-operative minimal residual disease^9^ and early cancer recurrence^10^. The gains in sensitivity and specificity, acquired through analytical methods like optimized Next Generation Sequencing (NGS), droplet-based digital PCR (ddPCR) or optimized qPCR, have allowed the detection of very small concentrations of ctDNA molecules in the bloodstream^11,12^.

Despite the great potential of ctDNA as a cancer biomarker, its application in clinical practice remains restricted to few indications. Amongst parameters that may limit the use of cfDNA and ctDNA in our routine practice, it is important to underline that the biological variables likely to modify the release of ccfDNA are not clearly comprehended. The origin of ccfDNA remains poorly understood and may imply several cell death processes^13^. Apoptosis has been proposed as a primary mechanism by which ccfDNA is released into circulation due to its distinctive fragment length of 140–180 bp, which mirrors the size of nucleosome-bound DNA fragments^14^. Necrosis and active release have also been described as a source of ccfDNA^13^. The ccfDNA fragmentation profile has been studied in different clinical situations^15–17^. Moreover, ccfDNA can be combined with diverse biological structures, including nucleosomes, extracellular vesicles (EVs; e.g., exosomes and virosomes) and neutrophile extracellular DNA trap (NETs) that could modify its integrity and physiological role^18^.

Moss et al. used a deconvolution methylation model in healthy subjects to show that ccfDNA mostly originates from white blood cells and erythrocyte progenitors^19^. Increased ccfDNA levels have been observed in various pathology such as cancers^20^, acute and chronic inflammatory diseases^21^, traumas^22^, and cardiovascular diseases^23^. The increase in ccfDNA concentration can also be observed in physiological processes such as physical activities^24^, pregnancy^25^ or obesity^26^. The analysis of ccfDNA concentration alone is therefore insufficient for the proper exploration of oncological processes. Analytical tests specifically targeting ctDNA by using cancer specific genetic and epigenetic alterations, have thus been developed^27–29^.

Although considerable efforts have been devoted to understanding the origin and the evolution of ccfDNA under various biological conditions, less attention has been paid to the daily dynamics of ccfDNA. Depending on the biological context, the estimated half-life of ccfDNA is comprised between 15 min and 1.5 h^30^. Understanding the potential daily variations of ccfDNA concentrations is critical to improve the reliability of ctDNA monitoring. Indeed, the circadian rhythmicity is a molecularly driven, individual clock. It generates cyclic changes through the activity of various compounds, with a periodicity of about a day^31^. This observation has led to chrono-pharmaceutical approaches to optimize regimen dosing and administration schedules^32^. Moreover, several studies have suggested that circadian rhythm could relate with the concentration of circulating nucleotides (miRNA and cfDNA) in subjects presenting with or without cancer^33–35^. The underlying mechanism is unknown. Moreover, a recent work by Diamantopoulou et al. has shown that the metastatic spread of breast cancer may accelerate during sleep^36^. They found that hormones implicated in the regulation of the circadian rhythm (such as melatonin, testosterone and glucocorticoids) may dictate the generation and the dynamics of circulating tumor cells, and as a consequence, that insulin may directly promote tumour cell proliferation in a time-dependent manner^36^.

The present study was designed to evaluate the influence of circadian rhythm on ccfDNA release in healthy subjects by focusing on its absolute quantification and fragmentation profile along the day. Such work could provide clues to determine the optimal timing for liquid biopsy sampling before its routine use in clinical practice. To reach this goal, we performed our analyses using ddPCR assays as well as BIAbooster system technology (miniaturized capillary electrophoresis) that allow for precise quantification of the total ccfDNA^17^. We first validated the repeatability and precision of the quantification method on commercial plasma samples from healthy subjects. We then analyzed the longitudinal ccfDNA concentration and fragmentation profile in a cohort of healthy subjects who underwent plasma sampling over a 24-h period. The impact of other variables on ccfDNA release such as weight, gender, creatinine concentration levels and ethnic origin was also evaluated.

## Materials and methods

### Subjects and procedures

**Figure 1:**
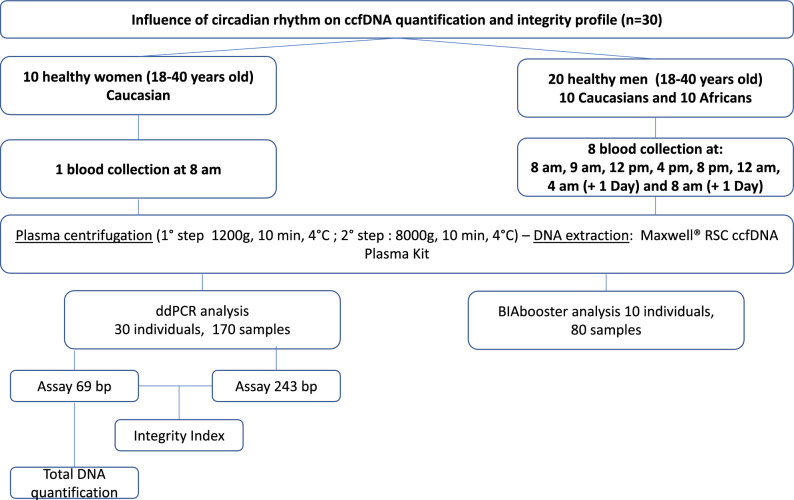
Workflow of the circadian study. In total, 20 healthy males and 10 healthy females were recruited. For each male, 8 blood samples were drawn. For each female, only one blood collection was drawn.

Longitudinal plasma samples collected from healthy subjects for the PSPRR cohort (previous cohort carried on in the Hospital Europeen Georges Pompidou (Plasma Soluble (Pro)Renin Receptor)) were available^37^. The study received the ethical approval from CPP Ile de France III (N°EUDRACT: 2010-A00365-34) ethics committee and all subjects provided with written informed consent. All processes were performed in accordance with the relevant guidelines and regulations. This healthy prospective cohort was composed of 20 healthy males and 10 healthy females for a total of 170 samples (Fig. 1). All subjects underwent blood sampling at 8 AM and only male subjects underwent longitudinal blood sampling over a 24 h period. Each male subject underwent 8 blood sampling at 8:00 AM, 9:00 AM, 12:00 PM, 4:00 PM, 8:00 PM, 12:00 AM, 4:00 AM (+ 1 Day) and 8:00 AM (+ 1 Day) using K2-EDTA tubes (Greiner Bio-One International, Cat. N°: 456043). Their meals were provided after blood sampling at 9:00 AM, 12:00 PM and 8:00 PM. Blood samples were collected in fasting conditions after 1-h rest in a semi recumbent position. To limit biases, subjects were asked to maintain a resting state throughout the study. The clinical characteristics of this healthy cohort are presented in the Supplementary Table 1. For one male, the weight and creatinine level data were not reported. Cortisol concentrations were measured using commercially available kits^37^. Secreted in a pulsatile fashion, it is known to display a circadian rhythm in adults were Cortisol levels are highest in the morning and subsequently decrease to a nadir in the evening^38,39^.

### Repeatability

To evaluate the repeatability of the quantification method, we used 2 cohorts of commercial plasma. Cohort 1 included samples from 13 healthy individuals from BIOPREDICT International and cohort 2 included 10 healthy individuals from the Etablissement Francais du Sang (EFS). Each plasma, previously stored at − 80 °C, was aliquoted three times (1 mL) and treated independently. For each aliquot of plasma, ccfDNA was extracted as described below ensuring that aliquots originating from the same plasma sample were treated on different experiments. These ccfDNA samples were quantified by 69 bp ddPCR assay. The workflow of this analysis is summarized in the Supplementary Fig. 1.

### Plasma ccfDNA preparation

The collection tubes (K2-EDTA) were processed within 4 h to prevent the destruction of white-blood cells at 1200g for 15 min at 4 °C. For each sample, 1 mL of plasma was aliquoted and stored at − 80 °C. Before extraction plasma were centrifugated a second time at 8000g for 10 min at 4 °C. The DNA was extracted using the Maxwell RSC ccfDNA Plasma Kit (Promega, Cat No./ID: AS1480) following the manufacturer’s instruction and was eluted in 62µL of elution buffer. The DNA concentration was measured from 2µL by Qubit 2.0 fluorometer (ThermoFisher Scientific) using the dsDNA HS Assay (ThermoFisher Scientific, Cat No. Q32851). Extracted DNA samples were stored frozen at − 20 °C before testing.

### ccfDNA analysis

#### Droplet-based digital PCR assays

We designed ddPCR assays to target the wild-type sequence of *KRAS* gene exon 2, codon 13. These assays allowed amplifying two sequences with lengths of 69 and 243 bp, which reflected the quantification of DNA fragments > 69 bp and > 243 bp, respectively. In both cases, a probe bearing a VIC fluorophore (λex 538 nm/λem 554 nm) detected the wild-type sequence of the *KRAS* gene (Supplementary Table 2).

#### ddPCR assays and DNA integrity index (DII) calculation

A total of 11 μL of ddPCR Supermix for Probes (2X) (No dUTP) (Ref: 1863024, BIO-RAD laboratories) was mixed with 1.1 µL of assay Mix solution (20X) containing 8 µM of forward (69 or 243 bp) and reverse primers, and 4 µM VIC Taqman® labeled-probes. The extracted plasma DNA was added to a final reaction volume of 22 μL.

The PCR assay mixes were prepared in a pre-PCR room to limit the risk of contamination. The probe bearing VIC-fluorophore was designed to be specific to the *KRAS* exon 2 WT allele. Emulsifications of DNA samples were generated according to manufacturer protocol (Ref: 1864002, QX200™ Droplet Generator, BIO-RAD laboratories). The emulsions were thermal cycled following different PCR programs described in the Supplementary Table 3. After completion, the emulsions were either stored at 4 °C or processed immediately to measure the end-point fluorescence signal from each droplet.

The concentration of ccfDNA in the plasma samples was determined as previously described^8^. Briefly, considering that an haploid genome equivalent (approx. 3 pg) is contained in a positive droplet, DNA concentrations were calculated by taking into account the volume of extracted plasma as well as the elution volume used for DNA extraction and the tested volume of DNA.

Since the number of positive droplets reveals the quantity of amplifiable target DNA, the fraction of amplifiable DNA in each sample could be determined. The number of DNA copies per well was determined using the QuantaSoft™ Analysis Pro Software (BIO-RAD laboratories). DII was defined as a ratio between the number of amplified copies of “*long” (243 pb)* and “*short” (69 pb)* DNA fragments. DII is calculated as below (Eq. 1):

1 $$DII WT= \frac{Quantity\,of\,DNA\,fragments\,corresponding\,to\,243\,bps\,fragment\,(WT) }{Quantity\,of\,DNA\,fragments\,corresponding\,to\,69\,bps\,fragment\,(WT)}$$

### BIAbooster analysis

The fragmentation profiles and concentration analyses of ccfDNA from 10 males (80 DNA samples) were also performed using BIAbooster System (ADELIS) as previously described^40^. The system is based on the principle of DNA fragment migration by capillary electrophoresis coupled to LED induced fluorescence (LEDIF) detectors. It allows performing size and concentration analyses of double stranded DNA with a sensitivity of 10 fg/μL in an operating time of 20 min. Based on the quantity of the different fragments obtained, a ratio highlighting the size distribution of the tested sample is calculated by BIAbooster technology as previously described^40^ (Eq. 2):

2 $$ratio = \frac{Detected\,Quantity\,of\,fragments\,with\,a\,size\,ranging\,between\,240-1650\,bps}{Detected\,Quantity\,of\,fragments\,with\,a\,size\,ranging\,between\,75-1650\,bps}$$

### Statistical analyses

In order to evaluate potential differences between DNA concentrations and integrity index at each collection time, paired non-parametric Friedman tests and Wilcoxon tests were performed. Mann Whitney test were performed for un-paired data. Post-hoc Dunn’s multiple comparison test was performed after significant Friedman test. Prism 9 software was used for statistical analysis.

## Results

### Evaluation of the repeatability of the method

**Figure 2:**
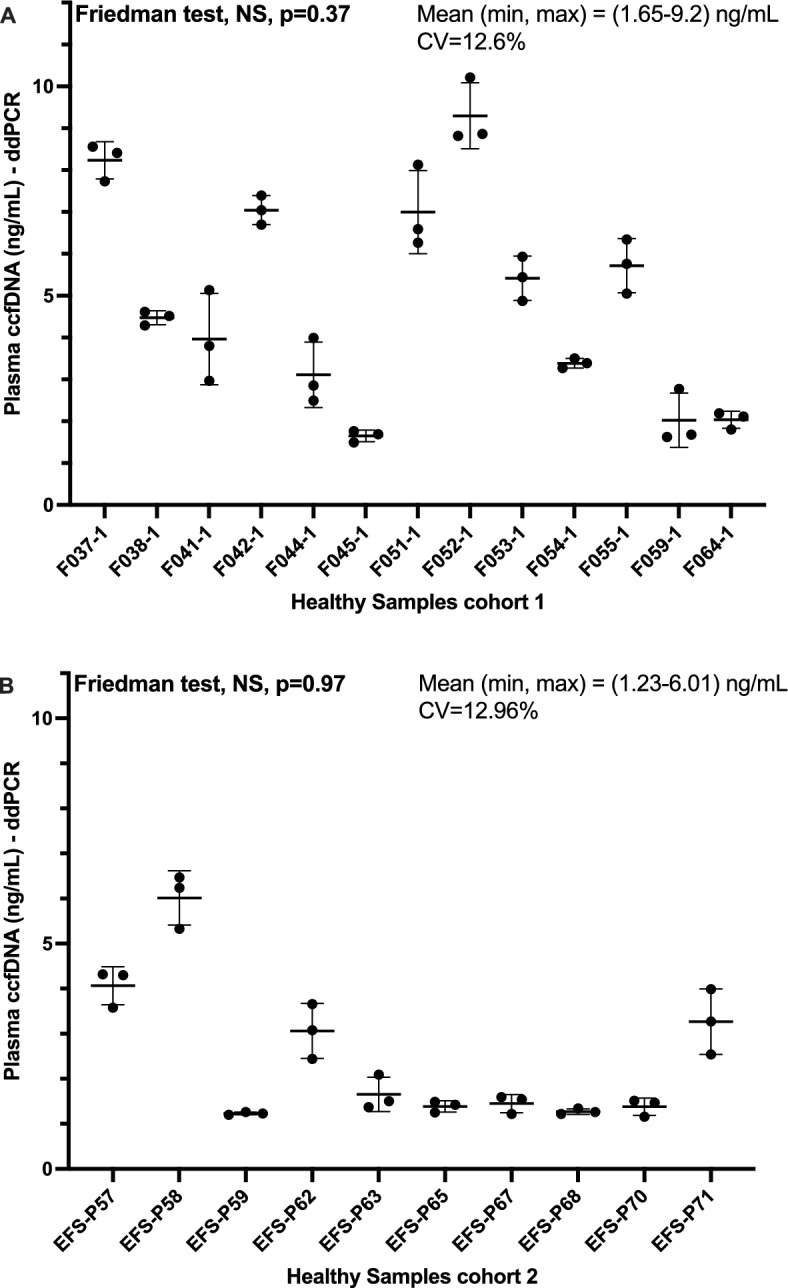
Repeatability of the pre-analytical and analytical process. The repeatability of the quantification method was evaluated using 2 independent cohorts of healthy plasma obtained from Biopredic international (**A**, n = 13) and Etablissement Francais du Sang (EFS, **B**, n = 10). In short, three aliquots were realized for each plasma. For each aliquot ccfDNA was extracted and its concentration determined by ddPCR (69 bp fragment assay). Aliquots originating from the same plasma sample were treated on different experiments.

The preliminary evaluation of the repeatability by ddPCR—69 bp assay was carried out. Figure 2 shows a representation of the variation of ccfDNA concentrations within replicates. The coefficient of variation was 12.6% in cohort 1 and 12.96% in cohort 2. No significant differences were observed between the plasma triplicates (Friedman test, p-value = 0.37 for cohort 1 and 0.97 for cohort 2). Both results suggest a repeatability of the pre-analytical and analytical methods. The ccfDNA concentrations in these cohorts were comprised between 1.2 and 9.2 ng/mL.

### Implication of the circadian rhythm on circulating DNA concentration

#### CcfDNA quantification by ddPCR

**Figure 3:**
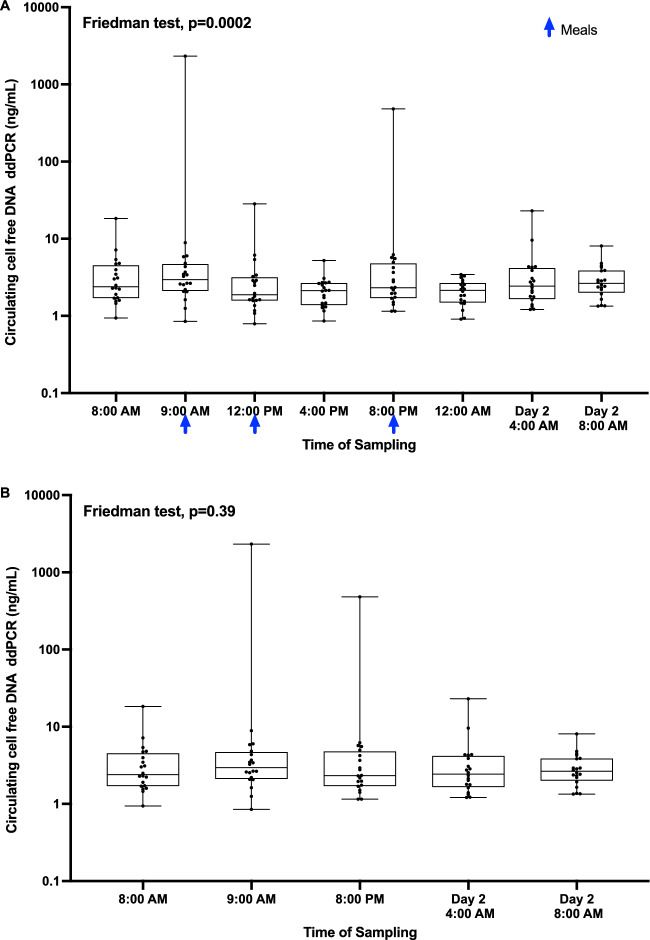
Impact of the circadian rhythm on ccfDNA release measured by ddPCR. (**A**) ccfDNA quantity (ng/mL) at each blood collection point over 24 h. (**B**) ccfDNA quantity (ng/mL) at each blood collection after exclusion of the post-prandial periods.

The 69 bp assay targeting the *KRAS* exon 2 codon 13 sequence was used to precisely quantify ccfDNA. Using this quantification, significant differences between ccfDNA concentrations were observed between blood collection times (Fig. 3A, Friedman non-parametric test, p-value = 0.0002). Post-hoc Dunn’s multiple comparison test was subsequently performed (Supplementary Table 4). The ccfDNA concentration was significantly decreased in samples drawn 3 to 4 h after meal ingestion (Time 12:00 PM, 4:00 PM & 12:00 AM). More specifically, ccfDNA concentration decreased between 9:00 AM and 12:00 PM and between 8:00 PM and 12:00 AM. It increased between 4:00 PM and 8:00 PM and between 12:00 AM and Day 1 + 4:00 AM. On the contrary, we observed no differences in ccfDNA concentrations between the samples drawn at distance from meal ingestion (Friedman non-parametric test, p-value = 0.39; Fig. 3B and Supplementary Table 5 for Post-hoc Dunn’s multiple comparison test). Normalization of ccfDNA against the value observed at 8am (time of arrival) did not modify the observed results (Supplementary Fig. 2A,B). As observed in this figure, there were some extreme values (2 values higher than 100ng/mL) of ccfDNA concentrations when compared to the commercial healthy cohorts (ie. cohorts 1 and 2) that can probably be attributed to pre-analytical sample treatment. However, these values did not modify the reported observations (data not shown).

The variation of cortisol concentration during the 24-h inclusion period, indicative of circadian rhythm, is shown in Supplementary Fig. 3. The ccfDNA and cortisol concentration were not correlated (Spearman r coefficient = 0.22). Between the healthy individual’s entrance in hospital (blood sampling at 8:00 AM) and the last sampling (the next day at the same time), ccfDNA concentration and proportion remained stable (respectively 3.87 and 3.12 ng/mL of plasma for 8:00 AM & Day 1 + 8:00 AM) (Supplementary Fig. 4).

#### CcfDNA quantification by BIAbooster system

**Figure 4:**
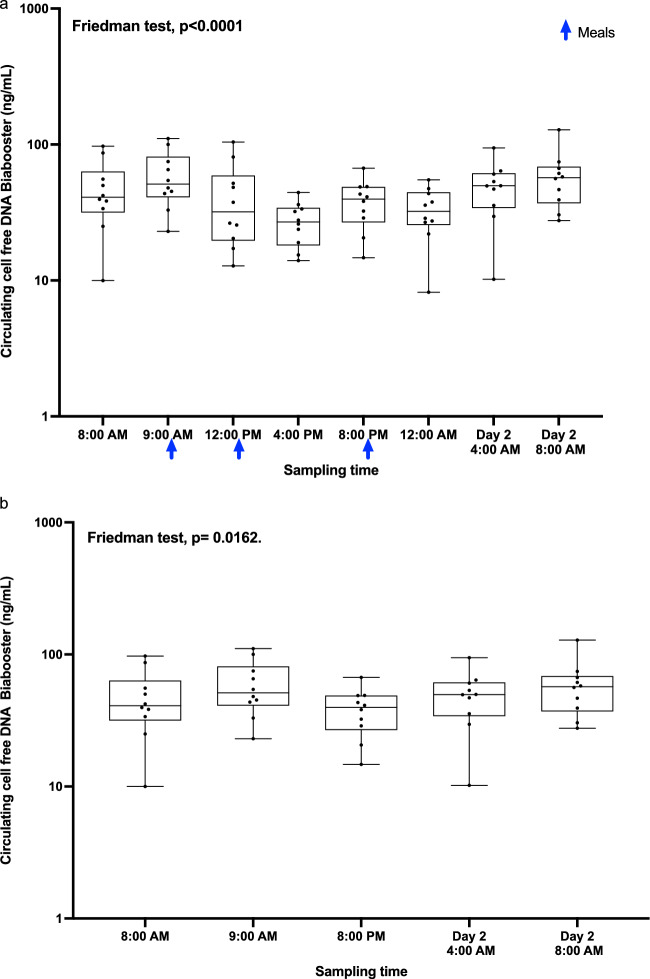
Impact of the circadian rhythm on ccfDNA release measured by BIAbooster system. (**a**) ccfDNA proportion at each timepoint over a 24-h period. (**b**) ccfDNA proportion for blood collection after exclusion of the post-prandial.

The ccfDNA concentration was also determined at each time-point and compared using the BIAbooster as previously described. As observed with ddPCR determination, we showed significant differences in ccfDNA concentrations between time-points of blood collection over 24 h period, related with meal ingestion (Kruskal–Wallis test, p-value = 1e−04) (Fig. 4a). Apart from the samples collected after meal ingestion (12:00 PM, 4:00 PM & 12:00 AM), we noticed no differences between other blood collection points (Kruskal–Wallis test, p-value = 0,068; Fig. 4b).

### Evaluation of the DNA integrity index over 24 h

**Figure 5:**
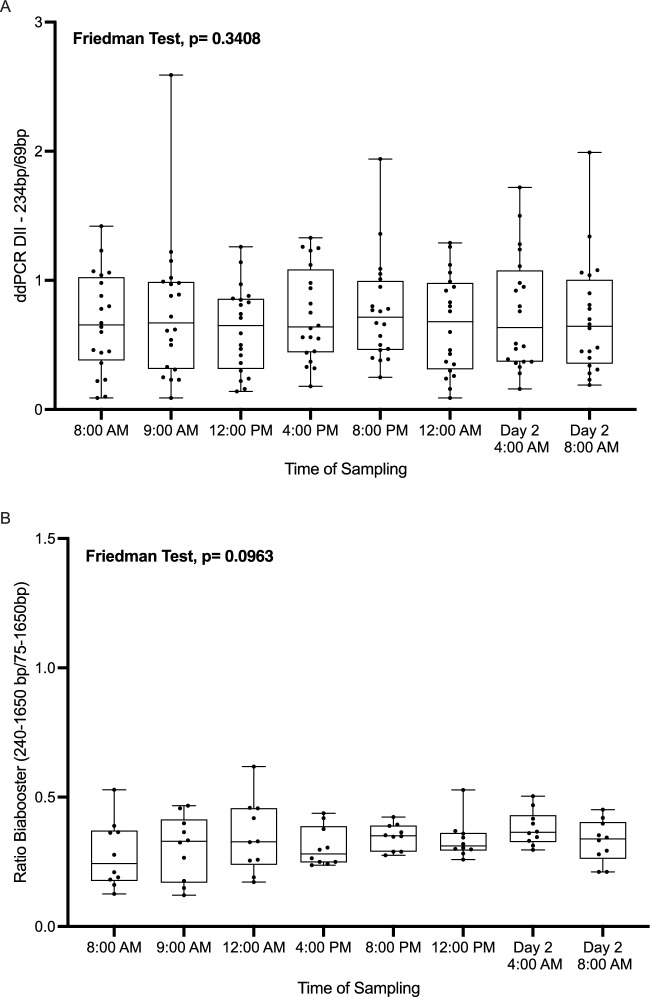
Influence of the circadian rhythm on ccfDNA integrity. (**A**) ccfDNA integrity index (DII) for each blood collection point over 24 h period. (**B**) ccfDNA ratio (Eq. 2).

Using the ddPCR − 243 bp assay, we quantified the corresponding DNA fragments. The DII index was determined for each DNA sampling-point following Eq. (2). Overall, no significant differences in ccfDNA integrity were observed between the different times-points (Friedman test, p-value = 0.34; Fig. 5A).

The integrity ratio calculated using the BIAbooster System showed similar results (Fig. 5B; Friedman test, p-value = 0.096).

### Influence of clinical parameters on ccfDNA concentration in healthy subjects

**Figure 6:**
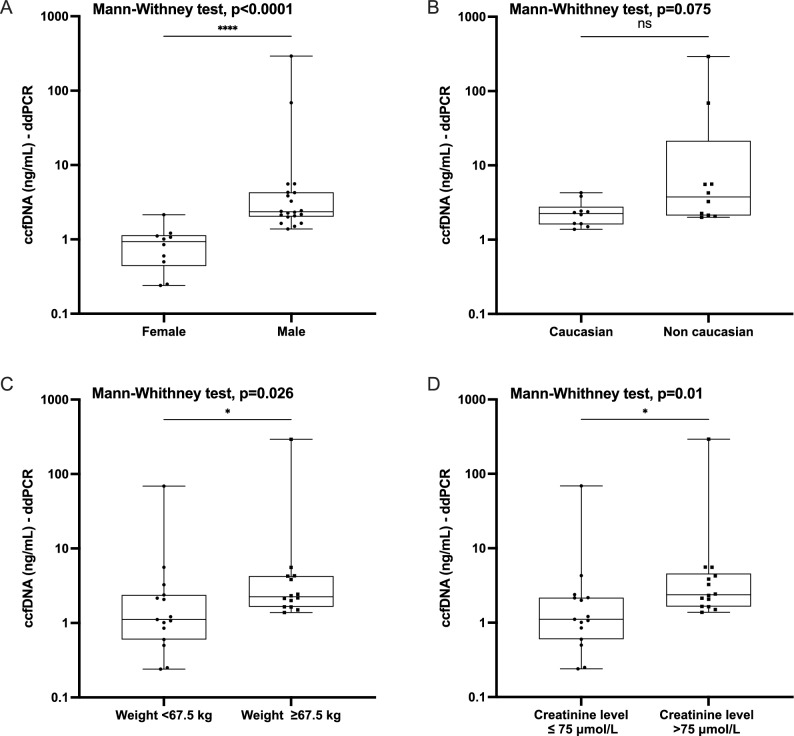
Influence of others clinical and biological parameters on ccfDNA release. (**A**) Gender. Comparison of plasma DNA concentrations between male and female. (**B**) Ethnicity. Comparison of plasma DNA concentrations between caucasian and non caucasian subjects. (**C**) Weight. The median weight of the cohort (female and male) was 67.5 kg. We defined two groups (< 67.5 kg and ≥ 67.5 kg). (**D**) Creatinine concentration. The median creatinine concentration of the cohort (female and male) 75 mol/L. We defined two groups (≤ 75 mol/L and > 75 mol/L).

Gender (n = 20 men & n = 10 women): Within the whole group, the ccfDNA concentration measured by ddPCR (69bp fragment) was higher in men (Mann Withney, p-value < 0.0001, Fig. 6A). This difference remains significant when excluding extreme ccfDNA concentration values (see above).

Ethnic status (n = 10 non-Caucasian men & n = 10 Caucasian men): In male subjects, we showed non-significant difference in ccfDNA concentrations according to the ethnical status (Mann Withney test, p-value = 0.075) Fig. 6B.

Weight (n = 15 with a weight ≤ 67.5 kg, n = 14 with a weight > 67.5 kg): The median weight of the whole cohort (women and men) was 67.5 kg. We therefore defined two groups, one with a weight ≤ 67.5 kg and one with a weight > 67.5 kg. The higher weight group was associated with higher ccfDNA concentrations (Mann Withney test, p-value = 0.026, Fig. 6C).

Creatinine (n = 15 with a creatinine concentration ≤ 75 µmol /L, n = 14 with a creatinine concentration > 75 µmol/L): Assuming the absence of renal failure in this population, the creatine level could be considered as a biomarker reflecting the muscular mass^41^. The median creatinine concentration level in this cohort (male and female) was 75 mol/L. We defined two groups, one with a creatinine concentration ≤ 75 mol/L and one with a creatinine concentration > 75 mol/L. The high creatinine concentration group showed higher levels of ccfDNA concentration (Mann Withney test, p-value = 0.01), suggesting a potential effect of muscular mass on ccfDNA release (Fig. 6D). Yet, creatinine level is often higher in males which may create bias in the interpretation of our results.

## Discussion

The ctDNA detection is a powerful marker that can be used for various applications including the detection of minimal residual disease and the diagnosis of cancer progression^42–44^. Several specific clinical applications are under evaluation in randomized control trials^45–47^. The fraction of ctDNA is small within cfDNA which can complicate its detection. To properly interpret those studies, it is crucial to pay close attention to the pre-analytical and analytical procedures, along with considering confounding factors associated with physiological conditions. The present trial is one of the rare studies that analyzed the link between the circadian rhythm and the detection of cfDNA.

In the first part of this study, we validated the absence of technical variability which ensured the pertinence of our biological data. We also confirmed that our technic benefited from a reproducible pre-analytical process starting from plasma preparation up to DNA extraction^48,49^.

Then we observed that the ccfDNA concentration and fragmentation profile remained stable during the day at each blood collection time (Figs. 3A, 4b, 5), apart from the samples drawn after meal ingestion. The circadian rhythm showed no interaction with ccfDNA release.

We observed a decrease in ccfDNA concentration after meal ingestion which could be related to a real postprandial effect on ccfDNA released. Yet, it could also be explained by technical bias. Indeed, difficulties to extract ccfDNA fragments have already been described after a meal absorption when the plasma is enriched in lipids and triglycerides^50^. In 2019, Meddeb et al. observed a decreased concentration of ccfDNA between 9:00 AM and 12:00 PM after meal ingestion^51^. In 2020, Lois Gardner et al. demonstrated that biomolecule corona of lipid nanoparticles may contain ccfDNA, suggesting an interaction between lipids and ccfDNA^52^.

We also noticed a significant difference in ccfDNA concentrations between women and men. However, the average weight between these groups was also different, which appears as a major confounding factor. Due to the small size of the present cohort, it was not possible to discriminate between gender or weight contribution on ccfDNA release. Such difference has not been observed for ctDNA in the ALGECOLS cohort^53^. The difference that we observed between the low and high weight groups (Fig. 6C) is in line with the literature^26,54,55^. Relationship between the adipocyte inflammatory process and the ccfDNA concentration in overweighted individuals has already been described^26^.

We also found a relation between creatinine and ccfDNA concentration level^41^. Yet all the female participant in our cohort were in the low creatinine group which may create bias. Further analyses, including the measurement of the muscular mass could be performed to clarify this observation.

The present work shows several limitations. Firstly, the cohort is small and only composed of 20 healthy male subjects. Another drawback is that the age of healthy individuals (18–35 years old) does not reflect the age of patients developing cancer (> 60 years old). Since aging, inflammatory processes and cell senescence may increase the ccfDNA concentration, a dedicated study should be performed on a larger cohort of healthy volunteers aged over 60 years old and include women. Further investigations on the potential postprandial effect on circulating DNA variation are also required.

In conclusion, circadian rhythm in healthy individuals did not clearly contribute to the variation in ccfDNA concentrations. However, these finding suggest that blood should be drawn ideally before meal ingestion to optimize the ccfDNA concentration. Further analyses are required to confirm those observations.

### Supplementary Information

Supplementary Figure 1.

Supplementary Figure 2.

Supplementary Figure 3.

Supplementary Figure 4.

Supplementary Table 1.

Supplementary Table 2.

Supplementary Table 3.

Supplementary Table 4.

Supplementary Table 5.

## Data Availability

The data that support the findings of this study are available from the corresponding authors VT or LB, upon reasonable request.
