# Supplementary material for: Circadian rhythm and circulating cell-free DNA release on healthy subjects
Source: Sci Rep. 2023 Dec 7;13:21675. doi: 10.1038/s41598-023-47851-w (PMC10709451; doi:10.1038/s41598-023-47851-w)
Supplement: Supplementary file 1 — Supplementary Figure 1. [file 41598_2023_47851_MOESM1_ESM.pdf]

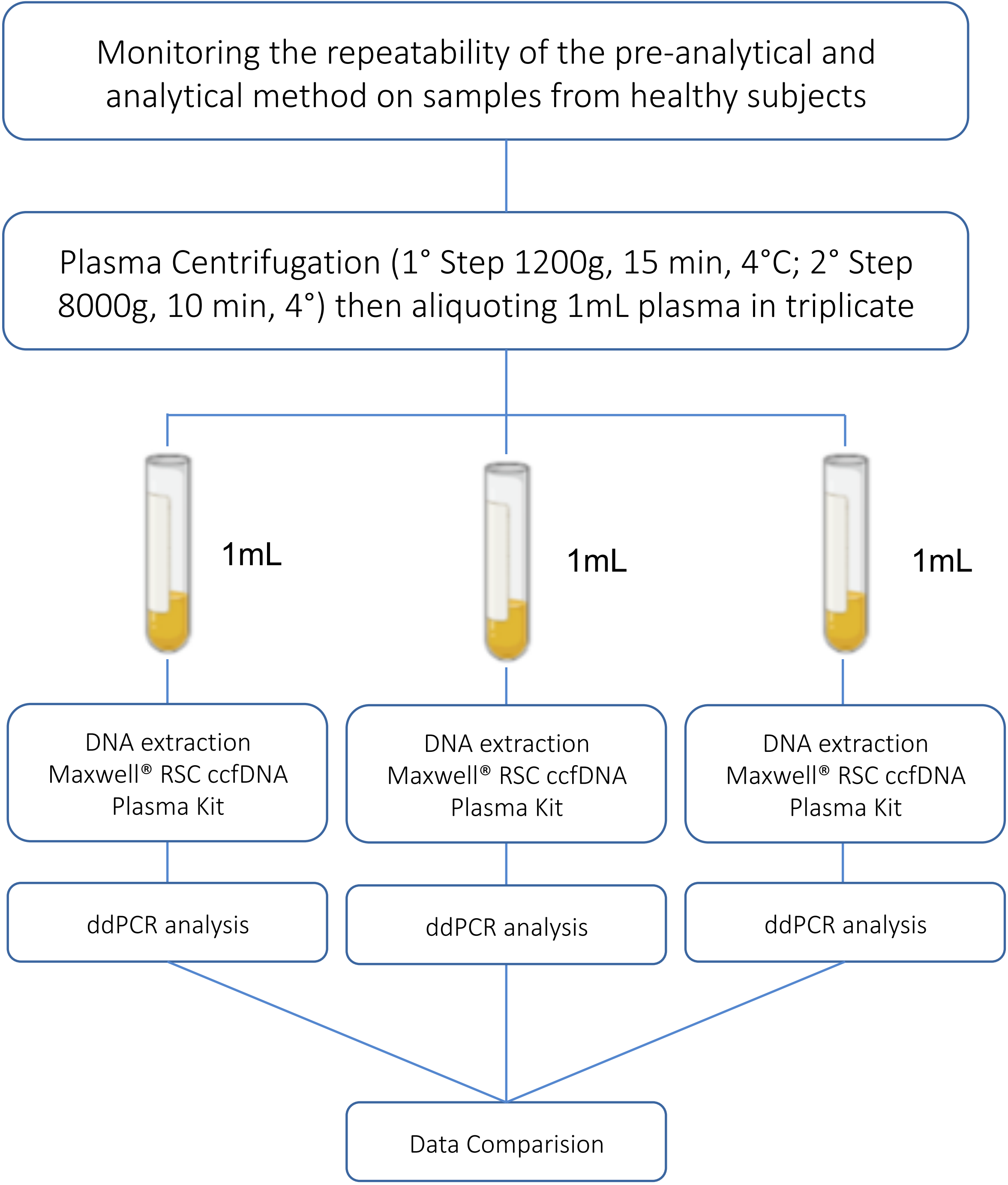

Supplementary Figure 1 : Analysis of the repeatability of the pre-analytical and analytical process applied on healthy plasma samples. The repeatability of the method has been evaluated on plasma samples from healthy subjects. For each subject, plasma has been aliquoted into 3 time 1 mL, then circulating cell free DNA has been extracted using the Maxwell@ RSC ccfDNA Plasma Kit and quantified by digital droplet-based PCR 69 bp assay.
