# Supplementary figures and images for: Circadian rhythm and circulating cell-free DNA release on healthy subjects
Source: Sci Rep. 2023 Dec 7;13:21675. doi: 10.1038/s41598-023-47851-w (PMC10709451; doi:10.1038/s41598-023-47851-w)

A

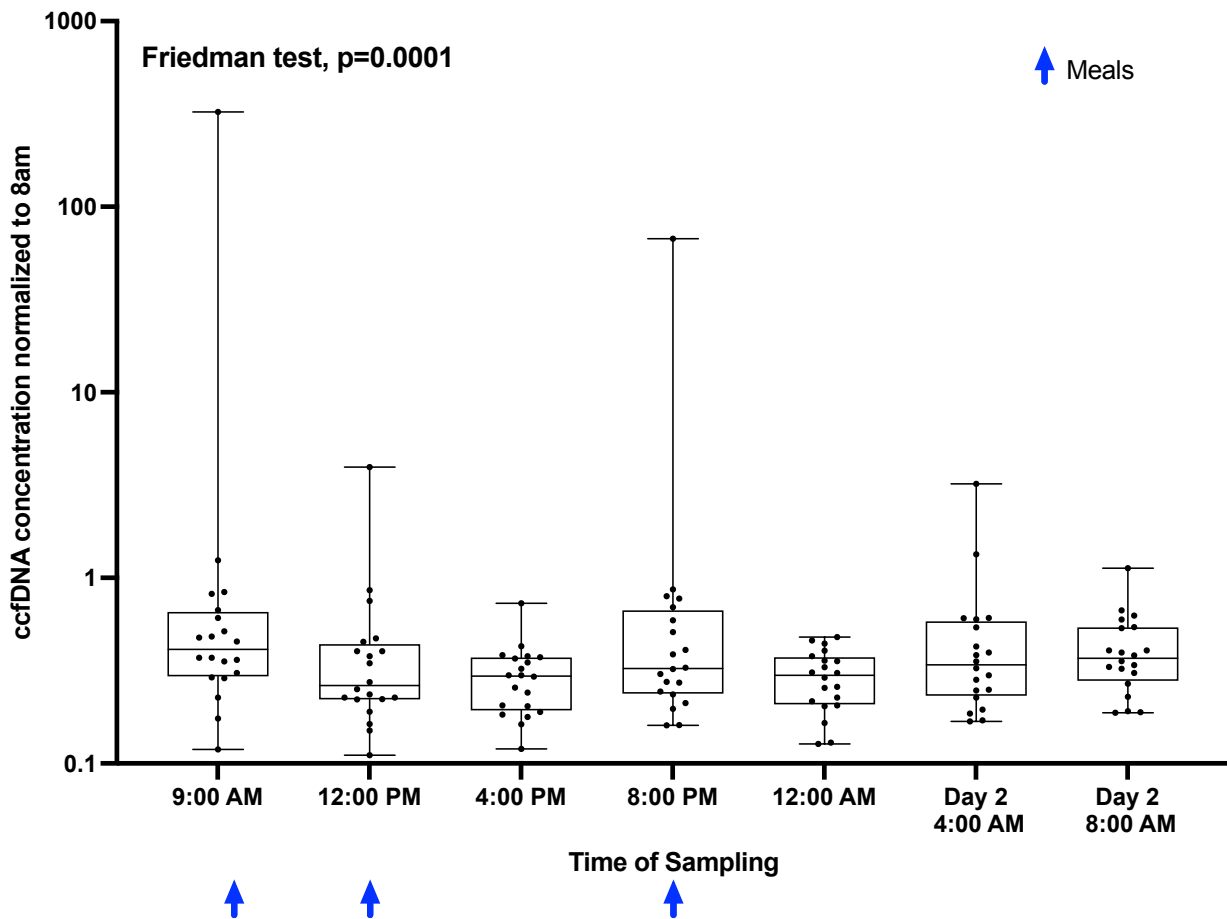

B

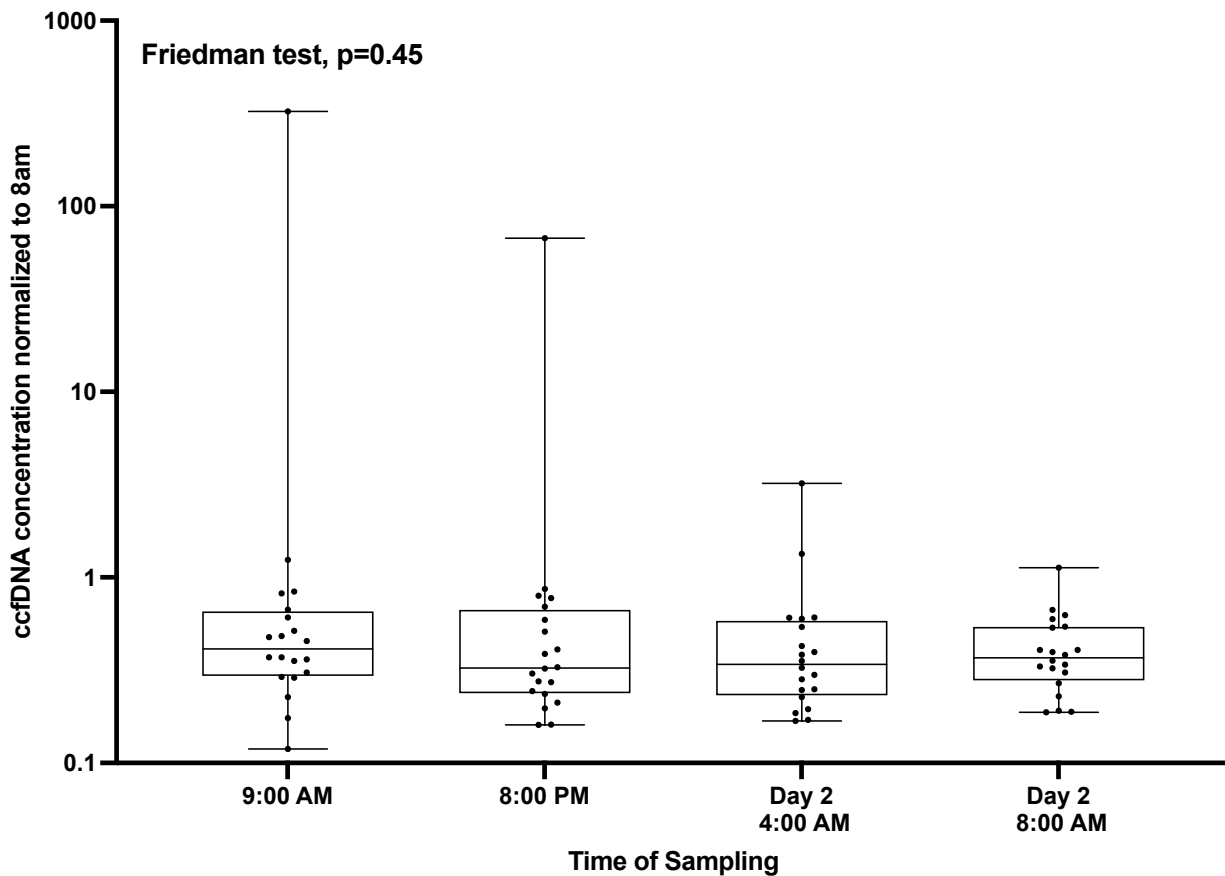

Supplement: Supplementary file 2 — Supplementary Figure 2. [file 41598_2023_47851_MOESM2_ESM.pdf]
