# Supplementary material for: Circadian rhythm and circulating cell-free DNA release on healthy subjects
Source: Sci Rep. 2023 Dec 7;13:21675. doi: 10.1038/s41598-023-47851-w (PMC10709451; doi:10.1038/s41598-023-47851-w)

Supplementary Figure 3: Variation of cortisol concentration (nmol / L) in blood during the 24h inclusion period for the 20 healthy males included in the study.

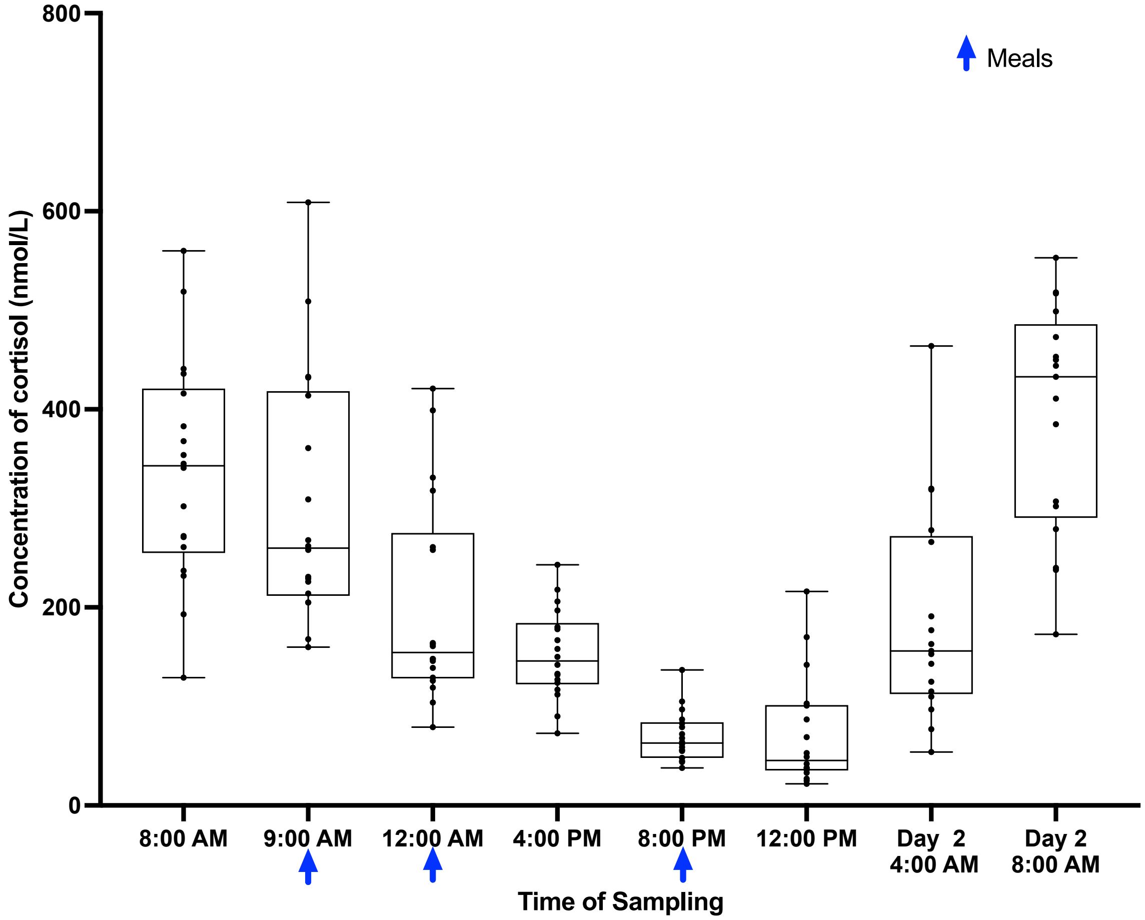

Supplement: Supplementary file 3 — Supplementary Figure 3. [file 41598_2023_47851_MOESM3_ESM.pdf]
