# Supplementary material for: Circadian rhythm and circulating cell-free DNA release on healthy subjects
Source: Sci Rep. 2023 Dec 7;13:21675. doi: 10.1038/s41598-023-47851-w (PMC10709451; doi:10.1038/s41598-023-47851-w)

Supplementary Figure 4: Comparison of the plasma circulating cell-free DNA concentrations between the first blood collection time (at 8:00 AM) and the last sampling time (at 8:00 AM of the next following day).

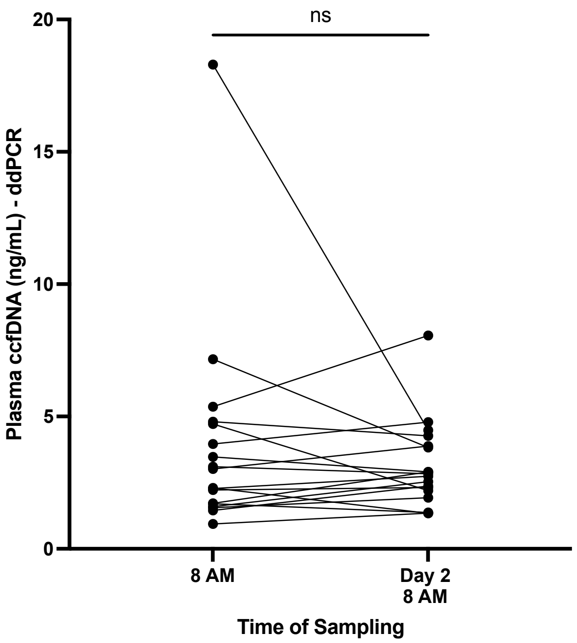

Supplement: Supplementary file 4 — Supplementary Figure 4. [file 41598_2023_47851_MOESM4_ESM.pdf]
