# Supplementary material for: Circadian rhythm and circulating cell-free DNA release on healthy subjects
Source: Sci Rep. 2023 Dec 7;13:21675. doi: 10.1038/s41598-023-47851-w (PMC10709451; doi:10.1038/s41598-023-47851-w)
Supplement: Supplementary file 5 — Supplementary Table 1. [file 41598_2023_47851_MOESM5_ESM.pdf]

Supplementary Table 1 : Basic clinical characteristics of healthy subjects included in this study referring main biological parameters: weight, creatinine, age, origin

Characteristics	Females, n=10[1]	Males, n=20[1]
**Weight**	57,8 [48,8, 66,3]	73,6 [68,9-91]
Unknown	0	1
**Creatinine**	61 [49, 72]	82,9 [66, 102]
Unknown	0	1
**Creatinine group**		
High creatinine concentration (>75)	0	14 (70%)
Low creatinine concentration (<75)	10 (100%)	5 (25%)
Unknown	0	1 (5%)
**Weight Group**		
High weight (> 67,5 kg)	0 (0%)	13 (65%)
Low weight (< 67,5 kg)	10 (100%)	6 (30%)
Unknown	0	1 (5%)
**Age**	26,1 [20.5-32,3]	27.1 [20.7-33,2]
**Origin**		
Caucasian	10 (100%)	10 (50%)
Not Caucasian	0	10 (50%)
**ccfDNA ng/mL**	0,9 [0,24-2.15]	20,59 [1,38-292,2]

[1]Statistics presented: Median (IQR); n (%)
