# Supplementary material for: Circadian rhythm and circulating cell-free DNA release on healthy subjects
Source: Sci Rep. 2023 Dec 7;13:21675. doi: 10.1038/s41598-023-47851-w (PMC10709451; doi:10.1038/s41598-023-47851-w)
Supplement: Supplementary file 6 — Supplementary Table 2. [file 41598_2023_47851_MOESM6_ESM.pdf]

Supplementary Table 2: Primers and probes sequences for the different digital PCR assays

	Sequence (5'>3')
**Reverse Primer**	TGTATCGTCAAGGCACTC
**Forward 69 bp**	GAAAATGACTGAATATAAACTTGTGGTA
**Forward 243 bp**	GCAGTCAACTGGAATTTTCA
**Probe KRAS WT (VIC)**	TGGTGGCGTAGGCA
**Probe KRAS MT (FAM)**	CTGGTGACGTAGGCA
