# Supplementary material for: Circadian rhythm and circulating cell-free DNA release on healthy subjects
Source: Sci Rep. 2023 Dec 7;13:21675. doi: 10.1038/s41598-023-47851-w (PMC10709451; doi:10.1038/s41598-023-47851-w)
Supplement: Supplementary file 7 — Supplementary Table 3. [file 41598_2023_47851_MOESM7_ESM.pdf]

Supplementary Table 3: PCR Programs

PCR Step	Temp (°C)	69 bp	243 bp	Cycle	Ramp rate (°C/min)
Enzyme activation	*50*	*2'*		*1*	
DNA denaturation	*95*	*10'*		*1*	*2*
Primers & Probe hybridization and elongation	*95*<br>*60*	*15''*<br>*30''*	*15''*<br>*1'15''*	*45*	*2*<br>*2*
Signal Stabilization	*98*<br>*12*	*10'*<br>*10'*		*1*	*2*
