# Supplementary material for: Circadian rhythm and circulating cell-free DNA release on healthy subjects
Source: Sci Rep. 2023 Dec 7;13:21675. doi: 10.1038/s41598-023-47851-w (PMC10709451; doi:10.1038/s41598-023-47851-w)
Supplement: Supplementary file 8 — Supplementary Table 4. [file 41598_2023_47851_MOESM8_ESM.pdf]

Supplementary Table 4: Intra-individual difference in ctDNA concentration along the day

Hours of sample drawn	Summary	Individual p value (Uncorrected Dunn's test)
8:00 AM vs. 9:00 AM	ns	0,1066
8:00 AM vs. 12:00 PM	ns	0,0933
8:00 AM vs. 4:00 PM	ns	0,0707
8:00 AM vs. 8:00 PM	ns	0,5186
8:00 AM vs. 12:00 AM	*	**0,0332**
8:00 AM vs. Day 2 4:00 AM	ns	0,4777
8:00 AM vs. Day 2 8:00 AM	ns	0,5613
**9:00 AM vs. 12:00 PM**	***	**0,0010**
**9:00 AM vs. 4:00 PM**	***	**0,0006**
9:00 AM vs. 8:00 PM	ns	0,3329
**9:00 AM vs. 12:00 AM**	***	0,0002
9:00 AM vs. Day 2 4:00 AM	ns	0,3662
9:00 AM vs. Day 2 8:00 AM	ns	0,3017
12:00 PM vs. 4:00 PM	ns	0,8973
**12:00 PM vs. 8:00 PM**	*	**0,0201**
12:00 PM vs. 12:00 AM	ns	0,6514
**12:00 PM vs. Day 2 4:00 AM**	*	**0,0169**
**12:00 PM vs. Day 2 8:00 AM**	*	**0,0239**
**4:00 PM vs. 8:00 PM**	*	**0,0142**
4:00 PM vs. 12:00 AM	ns	0,7469
**4:00 PM vs. Day 2 4:00 AM**	*	**0,0118**
**4:00 PM vs. Day 2 8:00 AM**	*	**0,0169**
**8:00 PM vs. 12:00 AM**	**	**0,0055**
8:00 PM vs. Day 2 4:00 AM	ns	0,9485
8:00 PM vs. Day 2 8:00 AM	ns	0,9485
**12:00 AM vs. Day 2 4:00 AM**	**	**0,0045**
**12:00 AM vs. Day 2 8:00 AM**	**	**0,0067**
Day 2 4:00 AM vs. Day 2 8:00 AM	ns	0,8973
