# Supplementary material for: Circadian rhythm and circulating cell-free DNA release on healthy subjects
Source: Sci Rep. 2023 Dec 7;13:21675. doi: 10.1038/s41598-023-47851-w (PMC10709451; doi:10.1038/s41598-023-47851-w)
Supplement: Supplementary file 9 — Supplementary Table 5. [file 41598_2023_47851_MOESM9_ESM.pdf]

Supplementary Table 5: Intra-individual difference in ctDNA concentration along the day after data normalization against ccfDNA concentration at 8am

Hours of sample drawn	Summary	Individual p value (Uncorrected Dunn's test)
**9:00 AM vs. 12:00 PM**	**	**0,0016**
**9:00 AM vs. 4:00 PM**	***	**0,0006**
9:00 AM vs. 8:00 PM	ns	0,3798
**9:00 AM vs. 12:00 AM**	***	**0,0003**
9:00 AM vs. Day 2 4:00 AM	ns	0,4208
9:00 AM vs. Day 2 8:00 AM	ns	0,3413
12:00 PM vs. 4:00 PM	ns	0,7697
**12:00 PM vs. 8:00 PM**	*	**0,0233**
12:00 PM vs. 12:00 AM	ns	0,6605
**12:00 PM vs. Day 2 4:00 AM**	*	**0,0192**
**12:00 PM vs. Day 2 8:00 AM**	*	**0,0281**
**4:00 PM vs. 8:00 PM**	*	**0,0104**
4:00 PM vs. 12:00 AM	ns	0,8836
**4:00 PM vs. Day 2 4:00 AM**	**	**0,0084**
**4:00 PM vs. Day 2 8:00 AM**	*	**0,0128**
**8:00 PM vs. 12:00 AM**	**	**0,0068**
8:00 PM vs. Day 2 4:00 AM	ns	0,9417
8:00 PM vs. Day 2 8:00 AM	ns	0,9417
**12:00 AM vs. Day 2 4:00 AM**	**	**0,0054**
**12:00 AM vs. Day 2 8:00 AM**	**	**0,0084**
Day 2 4:00 AM vs. Day 2 8:00 AM	ns	0,8836
